# Developing a Standardized Questionnaire for Measuring Older Adult’s Health and Well-Being in Kenya

**DOI:** 10.1093/geroni/igad137

**Published:** 2023-12-23

**Authors:** Lucy W Maina, Gloria Chepngeno-Langat, Samuel M Mwangi

**Affiliations:** Department of Sociology, Gender, and Development Studies, Kenyatta University, Nairobi, Kenya; African Population and Health Research Center, Nairobi, Kenya; Centre for Research on Ageing, University of Southampton, Southampton, UK; Department of Sociology, Gender, and Development Studies, Kenyatta University, Nairobi, Kenya

**Keywords:** Africa, Assessment, Indicators, Psychometric, Validation

## Abstract

**Background and Objectives:**

Policy and program interventions for older adults 60 years or older in Africa have consistently been undermined by a lack of vital data as they are usually excluded from nationally representative population surveys. The Health and Wellbeing of Older Persons in Kenya (HWOPs-1) study developed a standardized assessment questionnaire that can be used for periodic data generation. This study presents how the questionnaire was developed and examines its internal consistency and psychometric properties of the health module.

**Research Design and Methods:**

The development and validation of the HWOPs-1 questionnaire was a 3-step process. Step 1 was a review of 19 panel studies and 2 national level surveys followed by a wide consultation with key experts and stakeholders on aging. The 3rd step was validation of the questionnaire with a cross-section of a representative sample to test its applicability and adaptability in a mix of rural and semi-urban settings. The internal consistency and psychometric properties of the 3 subscales: functionality, disability, and quality of life were assessed using Cronbach’s (α) alpha and exploratory factor analysis, respectively.

**Results:**

Three subscales of functionality, disability, and quality of life showed high internal consistency with α = 0.94, 0.97, and 0.87, respectively. There were also consistent factor loadings above 0.3 across all the factors. Gender differences across the 3 scales from the results of *t* test were observed. Finally, weak but statistically significant correlations between the measures of well-being and risk factors for noncommunicable diseases were also observed from the analyses.

**Discussion and Implications:**

The indicators assessed have been used in settings outside Africa to measure health and well-being of older adults are adaptable and reliable enabling comparability across space and across studies. The questionnaire provides a framework for examining disease and disability burden and their determinants among older adults in Kenya or similar settings.

**Translational Significance:** Development and validation of instruments to measure health and well-being of any segment of population is crucial in ensuring that health determinants are accurately assessed cognizant of cultural and social contexts. Most general health and demographic surveys focus primarily on the reproductive age groups of 15–49. Assessment of older adults’ health and well-being in the African context is hampered by lacking cultural and context specific instruments. The questionnaire will be useful in providing accurate evidence on their health for the development of relevant policies, spot-on programming, and the improvement of health outcomes of the older adults in Kenya.

## Background and Objectives

The African continent is undergoing a demographic transition with the rapid increase in older adults aged 60 years or older. The United Nations and the African Union in their Policy Framework and Plan of Action on Ageing define older adults as those 60 years or older (HelpAge International, 2022), this definition is adopted in this study. In 2020, there were 74.4 million older people in the age group 60 years or older and this number is projected to triple by 2050. Furthermore, most countries in Africa will have at least one million older people in this age group (He et al., 2020). The importance of reliable data in comprehending the complex dynamics of population aging cannot be underscored. Indeed, accessing reliable scientific data enables the identification of areas for intervention and in monitoring and assessing the impact of policies. However, the availability of large, representative, and comparable data on aging and on issues affecting older people is almost nonexistent in Africa. With the youngest population and age distribution in 2022, Africa only had 5.5% of the global population of older adults in the 60 years or older age group and with a median age of 18.7 years compared with 26% and 41.2 years, respectively, for more developed regions of the world (United Nations, 2022). The very low proportion of older adults 60 years or older compared with the high concentration of children and young people has occasioned research bias with fewer studies targeting older adults’ realities. This has led to profound gaps in robust data as well as studies on the older population.

Formulation of social, health, and economic policies and interventions for older adults 60 years or older in Africa has consistently been undermined by a lack of data and vital statistics on the dynamics affecting the health and socioeconomic status of older adults. Reliable and comprehensive studies on older adults 60 years or older will enable adequate tracking of development agendas such as the Sustainable Development Goals (SDGs) for instance Goal 3 of the SDGs which aims at ensuring healthy lives and promoting well-being for all at all ages (United Nations, 2016). The World Health Organisation (WHO) has spearheaded a number of commitments and strategic frameworks on aging such as the Decade of Healthy Ageing 2021–2030 and other past initiatives such as the Global Strategy and Action Plan on Ageing and Health 2016–2020 (World Health Organisation, 2023). However, in order to set benchmarks and track progress, reliable indicators to measure progress are needed. Countries in Africa are normally excluded from tracking and evaluation of global agendas on aging due to a lack of reliable and comparable studies on aging and older adults. For instance, only eight countries in Africa were included in the 2015 Global Age Watch Index which assessed the well-being of older adults 60 years or older around the world across four domains; income security, health, employment, and enabling environment (HelpAge International, 2015). Thus, there is a need for African countries to develop data repositories on older adults 60 years or older and their health that are context-specific.

A number of instruments have been developed to assess the health and well-being of older adults 60 years or older in research studies and in practice. For instance, a systematic literature review identified 28 questionnaires used to assess mental well-being among older adults (Martín-María et al., 2021). The Integrated Care for Older People (ICOPE) framework by WHO assesses the intrinsic capacity impairment in clinical settings and among community-dwelling older adults (Hackert et al., 2021). The majority of these instruments are developed to assess and measure the psychological and mental well-being of older adults. However, they are not comprehensive and have ignored other domains of well-being including physical and social well-being. Furthermore, the instruments and measures are developed in cultural contexts that are different from Africa. There is a need to strengthen evidence gathering using reliable measurements and instruments that can enable monitoring and evaluation of the health and well-being of older adults 65 years or older (Amuthavalli Thiyagarajan et al., 2022; Macia et al., 2016).

Additionally, such evidence, if available in Africa, will inform and guide development planning and monitoring through periodic surveys such as the Demographic and Health Surveys (DHS) which are currently more focused on children, maternal, and reproductive health. The data may also form part of existing multi-country panels or repeat cross-sectional studies which monitor communicable diseases such as malaria and HIV/AIDS (ICF, 2023). Last, it is expected that the availability of data on older adults will lead to better representation in studies and publications. A systematic review to map the trend and types of research on aging in sub-Saharan Africa showed a slow growth in the number of peer-reviewed studies on older adults in the 80s and 90s and a more rapid increase in the past decade focusing mainly on HIV/AIDS and noncommunicable diseases (NCDs), with majority of the studies concentrated in South Africa followed by Nigeria and Ghana (Kalu et al., 2021). The bulk of the studies were cross-sectional involving small nonrepresentative samples whereas longitudinal studies were much fewer albeit still marred by a lack of large-scale representative samples. Overall, the review rated the methodological and reporting rigor of the studies to be of low to moderate quality particularly for qualitative studies highlighting the need for standardization in reporting and comparability of measures and indicators across studies and between countries (Kalu et al., 2022).

The World Health Organization Study on global AGEing and adult health (SAGE) is one such initiative designed to provide large-scale longitudinal and multi-country research on health and well-being. The SAGE was implemented in six countries, two of which are in Africa (Ghana and South Africa) and three waves of data collection were conducted between 2004 and 2018. The SAGE study has also spurred other mini-studies in Africa and ignited study networks such as the International Network for the Demographic Evaluation of Populations and Their Health in developing countries, the Medical Research Centre in Uganda, and the Africa Centre for Population and Health in South Africa (Kowal et al., 2012; World Health Organisation, 2007).

In response to the lack of data and vital statistics on the older population in Kenya, the Health and Wellbeing of Older Persons in Kenya: Tackling the Data Gaps and Needs’ (HWOPs-1) study sought to develop and validate a questionnaire to assess the health and well-being of older people in Kenya. This study presents the questionnaire developed from the study designed to build on the SAGE initiative and other similar efforts. The aim was to develop a comprehensive standardized assessment questionnaire with the potential to generate periodic data and evidence on the health and well-being of older adults 60 years or older in Kenya. The other objective of the study is to demonstrate data quality, reliability, and validity of the standardized questionnaire piloted in Kiambu County Kenya.

## Research Design and Methods

### Developing the Questionnaire

The questionnaire was developed through a review of studies that have been adopted from the U.S. Health and Retirement Study (HRS). There are over 19-panel studies globally that are part of the HRS-related family, and these were accessed through a platform developed to collate and harmonize the data (Lee et al., 2021). The HRS family of studies was selected because they have been designed to provide nationally representative multidisciplinary information on the social, economic, health, and well-being dynamics of an aging population. These studies have enabled the production of numerous research articles, reports, and policy documents which have changed and informed the understanding of aging dynamics in the countries where they are implemented in addition to informing policy and program directions (Fisher & Ryan, 2017; Steptoe, 2020). Other country surveys that informed the questionnaires were the DHS and the Kenya Integrated Household Budget Survey (KIHBS). The DHS and KIHBS have questions and indicators on sociodemographic status, living arrangements, livelihood activities, and economic and wealth status which have been piloted and adapted to the Kenyan social and economic context and are culturally appropriate in the context. In order to ensure that the questionnaire covers areas of interest for policy and programs, key government and non-governmental stakeholders on aging issues in Kenya were involved in the design. These were the Ministry of Labour and Social Protection, the Ministry of Health, the Kenya National Bureau of Statistics (KNBS), the National Council for Population and Development (NCPD), and HelpAge International.

The questionnaire consisted of two parts: the household roaster and the individual questionnaire. The household roster collected information on the household members’ demographic information including age, sex, and marital status, relationships of the household members, and level of schooling. Other information captured in the roaster included household amenities and housing characteristics such as sources of drinking water, toilet facilities, materials used to construct houses and ownership of assets in order to assess economic or wealth status of a household. The household was defined as a group of persons who live and eat together, whether or not they are related with caution not to be confused for a family similar to the DHS and KIHBS surveys to enable country-level and across-country comparison of household indicators. The individual questionnaire collected information on health and covered several life domains that are germane to older adults 60 years or older including family background, marital status and history, children and intergenerational relationships, work status and occupational history, health status descriptors, chronic conditions, lifestyle and health behavior, health care utilization and informal care support and community engagement. The questionnaire consisted of nine modules and the following are the content of each module.

#### Respondents’ background

This section covered questions on respondents’ age; migration history; religion; education and literacy; access and use of mass media, social media, internet, and mobile telephone; and access to financial services including mobile money.

#### Marital status and history

Information on current and previous marital status; duration of marital status; living arrangement with spouse; polygamy; and age at first marriage.

#### Children and family history

The questions on the older adults’ children included information on both biological and non-biological children, survival and current living arrangements of the children, and number of grandchildren they had. The survival status of the older adults’ parents and siblings was collected to document existing family networks.

#### Work status, work history, and benefits

Information on the livelihood activities that the older adults engaged in, their current and previous employment, their main sources of livelihood, receipt of statutory benefits, and old age benefits such as pension or retirement benefits were collected.

#### Health status

Questions on health status formed the bulk of the questionnaire and these were self-reported and covered disease diagnosis, health care access and utilization, functional limitations and disability, falls/injuries, and quality of life (QoL). Questions on diagnosed chronic illnesses and conditions covered diabetes mellitus, hypertension, arthritis, chronic lung disease, angina, stroke, cancer, asthma, depression, and dementia. This study assesses the functional limitations, disability, QoL, and chronic conditions subscales.

#### Lifestyle and health behavior

Information collected on risk factors included smoking, alcohol consumption, physical activities, and diet.

#### Health care utilization

The section dwelt on questions regarding health care seeking (in and outpatient care), reasons for seeking health care, ability/inability to access health care, and sources of health care costs (including health insurance).

#### Informal care and support and community engagement

Questions on informal care included intergenerational care and support, and sources of informal care, met and unmet need for informal care. Community engagement covered questions on older adults’ engagement and participation in formal and informal community activities, and participation in organized social groups.

#### Elder abuse, neglect, and mistreatment

This module captured the experience of physical, financial, emotional, or sexual abuse and injuries resulting from the abuse. To assess neglect, the questionnaire explores the necessity for help with various tasks relating to instrumental activities of daily living (IADL), activities of daily living (ADLs), and personal care immediately following abuse, neglect, and mistreatment.

## Methodology and Approach in the Development and Piloting of the Health and Well-Being of Older Persons in Kenya: Tackling the Data Gaps and Needs’ (HWOPs-1) Questionnaire

The development and validation of the questionnaire involved a three-step process. The first step entailed a desk review of the HRS family of studies and a panel of experts to assess the extant instruments for measuring older adults’ health globally as part of isolating significant indicators. Additionally, using the HRS database (The Center for Economic and Social Research, 2015), a review of existing published literature based on the family of HRS studies on older adults health was undertaken to isolate general and unique indicators of health with a careful analysis of their applicability in the context. Articles reporting on studies conducted in low- and middle-income countries and on the broad subject of health were first selected and this yielded 594 peer-reviewed articles, these were further selected to include only studies on methodology resulting in 25 articles for review. This process allowed for assessing and selecting indicators or measures that emerged critical in measuring health in the African context. In order to ensure a comprehensive coverage of all possible indicators and items, in the second step, consultations were undertaken with key experts and stakeholders on aging and health matters in the country ranging from government ministries and agencies such as the Ministry of Labour and Social Protection-State Department of Social Protection, Ministry of Health, NCPD, KNBS and Help Age International. Through a series of participatory workshops with stakeholders and researchers including representatives of older adults, the emerging questionnaire was scrutinized and refined informed by experiences and practice to ensure the inclusion of all key factors that have a bearing on individual and specifically older adults’ health.

The third step of the study entailed validation of the developed questionnaire through field study. A cross-sectional design was used to test the applicability and adaptability of the questionnaire in a rural and semi-urban region of the country. Kiambu County was purposively selected due to its mixed rural and urban character, proximity to the city of Nairobi as well as its cosmopolitan nature and varying climate and topographical terrain. The selection of participants employed probability sampling techniques allowing an unbiased sample estimation using a master sampling frame usually used in national studies by the KNBS. A multi-stage sampling method was applied to select a statistically sufficient number of Enumeration Areas (EAs) required to achieve a desired sample based on the estimated proportions of older adults 60 years or older to the larger population. To raise at least 800 older adults for the study, 82 EAs were required, and these were randomly selected from the list of 4,946 EAs in Kiambu County based on the probability of acquiring the desired sample of 800 older adults. To facilitate the process of listing, cluster maps and their GPS coordinates from the KNBS were procured. A listing exercise then followed where all households that had persons 60 years or older in the selected 82 EAs were identified resulting in 2,892 eligible individuals (i.e., the target population). Proportionate stratified random sampling was then used to select households enabling a proportionate size of respondents to be drawn from each EA relative to size followed by a random selection of participants from each household with an older adult. To cater for non-response and reflect the country’s own experience with non-response (typically under 10% in most developing countries), a 10% proportion was added to the sample of 800 (0.1 × 800) yielding an additional 80 participants.

The study protocols were reviewed by the Kenyatta University Ethical Review Committee and the Scientific Steering Committee (Ref. No. PKU/8691934). The questionnaire was interviewer-administered using electronic tablets on the surveyCTO platform. Experienced field teams were recruited to collect the data after undergoing in-class and field training to ensure familiarity with the data collection instruments, compliance with study methodology, ethics and field logistics. To monitor quality during fieldwork, regular spot checks were conducted in the field and data was analyzed as it was uploaded daily to the server to monitor trends and inconsistencies. The response rate was 89%.

### Characteristics of the Study Participants

Table 1 shows the percentage distribution of the sociodemographic characteristics of the participants. A total of 783 older adults completed the interview, the average age was 74 years, and the majority of the participants were women (65%). Table 1 compares the men and women respondents, women were more likely to be older (74.6 vs 72.8 years), widowed (55% vs 13%) divorced (10% vs 4%), or never married (8% vs 2%), and with less education (35% had attained no education compared with only 8% of the men). The majority of older adults depended on their own work or livelihood activities (51%) with only 3% relying on pension as the main source of income. A higher proportion of women depended on their children (38%) compared with men (14%). At the age of 50 years, more women were looking for work (46%) compared with men (23%) while slightly more than half (54%) were still in employment.

**Table 1.** Percentage Distribution of the Sociodemographic Characteristics of Study Participants

Sociodemographic variable	Men (%)	Women (%)	Total (%)	Test statistic
Sex	34.7	65.3	100.0	
Mean age	72.8	74.6	74.0	*t* = 16.5, *p* < .001
Marital status***				χ^2^ = 211.1, *p* < .001
Married	80.5	26.4	45.2	
Divorced/separated	4.4	10.0	8.1	
Widowed	13.2	55.4	40.7	
Never married	1.8	8.2	6.0	
Level of education***				χ^2^ = 126.4, *p* < .001
No education	8.1	34.6	25.4	
Primary	57.4	43.3	48.2	
Secondary	24.3	6.7	12.8	
Collage or higher	6.3	1.8	3.3	
Other	4.0	13.7	10.3	
Employment status @ 50 years***				χ^2^ = 70.7, *p* < .001
Retired	7.7	5.3	6.1	
Employed	54.4	26.2	36.0	
Looking for work	23.5	46.2	38.3	
Laid off	10.7	15.5	13.8	
Other	3.7	6.9	5.8	
Main source of livelihood***				χ^2^ = 56.9, *p* < .001
Own work	53.7	34.3	41.0	
Own savings/investment	10.7	9.6	10.0	
Pension/retirement	6.6	1.4	3.2	
Support from children	14.3	38.2	29.9	
Support from others	14.7	16.6	16.0	

*Note*: *p* Value significance level ***<.001.

### Analyzing the Psychometric Properties of the HWOPs-1 Questionnaire

To assess the psychometric properties of the HWOPs-1 questionnaire, an analysis to determine the internal consistency and convergent validity was conducted. The Cronbach’s alpha (α) test was used to assess the internal consistency. To assess validity, a comparison of the prevalence of the two most commonly reported non-communicable chronic diseases among older persons namely diabetes and hypertension with existing research was done. The other method used to evaluate validity was to assess the correlation between the three scales (functionality scale, QoL scale, and disability scale), and various indicators. These were correlated with other health indicators (global self-rating of overall health, happiness, and life satisfaction) and with socioeconomic indicators (lifestyle behaviors associated with poor health or well-being outcomes associated with aging) known to have a strong association with health and well-being. Following is a description of the health indicators and how these were operationalized.

A *functionality scale* that assesses the ability to perform IADL was created by aggregating 18 items that assess functional status across seven subscales namely mobility (2 items), self-care (3 items), pain and discomfort (3 items), cognition (2 items), interpersonal relationships (4 items), sleep and energy (2 items), and affect (2 items). The participants were asked to self-rate the difficulty they had in the 30 days preceding the interview with aspects relating to these seven subscales on a 5-point scale ranging from no difficulty to extreme difficulty. The summed value which ranged from 18 to 90 with a higher value indicating greater difficulty was further grouped into three categories mild, moderate, and severe difficulty with functionality.

The *disability scale* used the 24-item World Health Organization Disability Assessment Schedule (WHODAS II) which evaluates difficulty specific to ADLs and IADLs. The questions included difficulty as a result of a health condition, with sitting for long periods, walking, standing up, climbing, grip, participation outside the home, concentrating, moving around the house, eating, using the toilet, and dressing. These activities were assessed on a 5-point Likert scale. The values ranged from 0 meaning no difficulty at all to 120 indicating inability to perform ADLs. and after summing, the values were grouped into three categories for the purposes of analysis.

The *QoL scale* consists of seven items and captured participants’ satisfaction with (i) everyday life, (ii) with enough money to meet their needs, (iii) with their health, (iv) with personal relationships, (v) with the conditions of their living place, (vi) with performing ADLs and (vii) with life as a whole. The participants rated their satisfaction on a 5-point scale from *Very satisfied*, *Satisfied*, *Neither satisfied or dissatisfied*, *Dissatisfied*, and *Very dissatisfied*. The 7 items were summed and categorized into three groups.

Other indicators used are the global health and QoL measures which asked the participants; *“In general, how would you rate your health today, would you say your health is 1 Very bad, 2 Bad, 3 Moderate, 4 Good, 5 Very good’?* and ‘*How would you rate your overall quality of life’? Is it 1 Very bad 2 Bad, 3 Moderate, 4 Good, or 5 Very good?”*

To compare these health indicators with areas that are known to greatly affect health, well-being, and QoL, two indicators were selected: vision and feeding/chewing food. The participants were asked to rate the level of difficulty, on a 5-point scale, their ability to see and recognize an object at arm’s length for example, reading, and if they had any problems with their mouth and/or teeth, including problems with swallowing. Socioeconomic status is known to have a bearing on health outcomes, therefore the correlation between health indicators and level of education and type of employment the participant was engaged in prior to attaining 50 years was assessed.

To verify the psychometric properties of the HWOPs-1 questionnaire, exploratory factor analysis was conducted for each scale: functionality, disability, and QoL. Five (5) principal factors were extracted for both functionality and disability subscales which have 18 and 24 items, respectively, while three (3) factors were extracted for the QoL subscales with 8 items. All items had intercorrelations of less than 0.8, and thus were all retained in the analyses.

## Results

Results from Exploratory Factor Analysis (Table 2) show consistent factor loadings above 0.3 in two of the three subscales. In both functionality (i.e., all 18 items) and disability (i.e., all 24 items) subscales, all items had factor loadings above 0.3 on all five factors. However, in the QoL subscale which had 7 items, one of them had factor loadings below 0.3 on all three factors. These findings point to a strong internal consistency of the disability and functionality scales, but the QoL subscale had items with low intercorrelation. As reported earlier, the participants had reported higher scores in both disability and functionality scales and relatively lower scores in QoL scores, thus explaining the same pattern supported by exploratory factor analysis.

**Table 2.** Exploratory Factor Analysis Results for Disability, Functionality, and Quality of Life Scales

HWOPs-1 scale	Factor loading				
	1	2	3	4	5
Disability scale					
Difficulty sitting for long periods e.g., 2 hr (last 30 days)	0.62	–0.15	0.12	0.19	0.20
Difficulty in walking 100 meters (last 30 days)	0.81	–0.15	–0.02	–0.03	0.15
Difficulty in standing up from sitting (last 30 days)	0.77	–0.27	0.09	0.06	0.37
Difficulty standing for long periods (last 30 days)	0.73	–0.45	–0.05	–0.02	0.11
Difficulty with climbing one flight of stairs or steep slope without resting (last 30 days)	0.69	–0.36	–0.18	–0.13	0.03
Difficulty picking up things with your fingers (last 30 days)	0.66	0.22	–0.08	–0.03	0.13
Difficulty taking care of household responsibilities (last 30 days)	0.78	0.00	–0.18	0.12	–0.16
Difficulty in joining community activities (last 30 days)	0.80	0.04	–0.13	0.04	–0.14
Difficulty extending arms above shoulder level (last 30 days)	0.71	0.09	–0.08	0.05	0.07
Difficulty concentrating on something for 10 min (last 30 days)	0.68	0.22	–0.07	0.09	0.01
Difficulty walking a long distance e.g., 1 kilometer (last 30 days)	0.78	–0.39	–0.16	–0.08	–0.04
Difficulty in bathing/washing whole body (last 30 days)	0.78	0.40	–0.16	0.13	–0.01
Difficulty in getting dressed (last 30 days)	0.77	0.45	–0.21	0.18	0.02
Difficulty in day-to-day work (last 30 days)	0.79	–0.18	–0.12	0.06	–0.25
Difficulty in carrying things (last 30 days)	0.73	–0.29	–0.20	–0.06	–0.17
Difficulty with moving around inside home (last 30 days)	0.79	0.24	0.01	0.00	0.11
Difficulty in eating (last 30 days)	0.54	0.15	0.07	0.08	0.05
Difficulty getting up from lying down (last 30 days)	0.74	–0.06	0.12	–0.04	0.08
Difficulty getting to and using the toilet (last 30 days)	0.72	0.24	0.14	–0.03	0.04
Difficulty getting to where you want to go using private transport if needed (last 30 days)	0.69	0.20	0.14	–0.40	–0.01
Difficulty getting to where you want to go using public transport if needed (last 30 days)	0.81	0.16	0.13	–0.38	–0.08
Difficulty getting out of home (last 30 days)	0.83	0.17	0.11	–0.15	–0.04
How much emotionally affected by health condition (last 30 days)	0.71	–0.09	0.39	0.16	–0.19
Overall, how difficulties interfere with life	0.78	–0.17	0.38	0.22	–0.18
Functionality scale					
Difficulty with moving around (last 30 days)	0.76	0.18	–0.29	0.04	–0.03
Difficulty with vigorous activities (last 30 days)	0.58	0.34	–0.20	0.13	–0.01
Difficulty with self-care (last 30 days)	0.84	–0.13	–0.31	–0.36	–0.03
Difficulty with taking care and maintaining general appearance (last 30 days)	0.86	–0.16	–0.24	–0.30	0.01
Difficulty with staying with yourself for a few days (last 30 days)	0.71	–0.08	–0.15	–0.05	–0.02
Bodily pains or aches (last 30 days)	0.68	0.47	–0.17	0.29	–0.06
Bodily discomfort (last 30 days)	0.74	0.44	–0.18	0.28	–0.10
Difficulty in daily life due to aches, pains, or discomfort (last 30 days)	0.81	0.06	–0.29	–0.19	–0.04
Difficulty with concentrating or remembering something (last 30 days)	0.72	–0.09	0.03	0.08	0.01
Difficulty with learning a new task (last 30 days)	0.76	–0.16	0.01	0.08	0.03
Difficulty with personal relationships/participation in community activities (last 30 days)	0.75	–0.32	0.04	0.12	0.00
Difficulty with dealing with conflicts and tensions (Last 30 days)	0.73	–0.44	0.15	0.11	–0.01
Difficulty with making new friends or maintaining current friends (last 30 days)	0.70	–0.54	0.16	0.16	0.03
Difficulty with dealing with strangers (last 30 days)	0.64	–0.51	0.21	0.15	0.02
Problem with sleeping (last 30 days)	0.56	0.37	0.33	–0.05	0.34
Problem due to not feeling rested and refreshed during the day (last 30 days)	0.66	0.31	0.26	–0.10	0.41
Problem with feeling sad, low, or depressed (last 30 days)	0.59	0.28	0.54	–0.13	–0.27
Problem with worry or anxiety (last 30 days)	0.60	0.23	0.53	–0.16	–0.27
Quality of life scale					
Have enough energy for everyday life	–0.60	0.24	0.22	—	—
Have enough money to meet needs	–0.56	0.01	0.45	—	—
Satisfaction with health	0.83	–0.24	0.11	—	—
Satisfaction with ability to perform daily living activities	0.88	–0.29	0.19	—	—
Satisfaction with personal relationships	0.62	0.09	0.10	—	—
Satisfaction with conditions of living space	0.67	0.53	–0.01	—	—
Overall satisfaction with current life	0.82	0.23	0.09	—	—

Table 3 shows the summary statistics and the descriptive analysis of the health scales: functionality scale, disability scale, QoL scale, and the two global indicators of health and QoL compared across sex. The *t* test was used to examine the differences in means between the sexes. Women on average reported higher scores on the functionality scale (44.3, *SD* 14.6 vs 35.8, *SD* 14.2) and on the disability scale (56.5, *SD* 22.4 vs 45.0, *SD* 20.1) indicating that they were more likely to report poorer health compared with men. Conversely, men reported slightly higher scores compared with women on the QoL scale (23.0, *SD* 3.3 vs 22.7, *SD* 3.5) meaning they rated their QoL poorer than women although this difference was not statistically significant.

**Table 3.** Summary Statistics for the Measurement Scales by Sex

Health measurement scales	No. of items	Range	Min	Max	Mean (*SD*)	Test Statistic
Functionality scale***						*t* = –7.91, *p* < .001
Men	18	18–90	18	83	35.8 (14.2)	
Women	18	18–90	18	90	44.3 (14.6)	
Total	18	18–90	18	90	41.3 (15.0)	
Disability scale***						*t* = –7.12, *p* < .001
Men	24	0–120	24	118	45.0 (20.1)	
Women	24	0–120	14	120	56.5 (22.4)	
Total	24	0–120	14	120	52.5 (22.3)	
Quality of life scale						*t* = 0.94, *p* > .1
Men	7	7–35	13	29	23.0 (3.3)	
Women	7	7–35	12	30	22.7 (3.5)	
Total	7	7–35	12	30	22.8 (3.4)	

*Notes*: SD = standard deviation. *p* Value significance level ***<.001.

The multi-item scales to assess functionality, disability, and QoL (functionality scale, disability scale, and QoL scale, respectively) had very good internal consistency demonstrated by Cronbach alpha of 0.94, 0.97, and 0.87, respectively.

Table 4 compares the prevalence rate for commonly reported NCDs among adults with other studies in Africa where data has been disaggregated by age. In the HWOPs-1 study, 11.7% of participants reported to have been diagnosed with diabetes. Although slightly lower, the prevalence of diabetes captured in the HWOPs-1 study was comparable to other studies conducted in Kenya and Ethiopia (Gebreegziabiher et al., 2020). The findings on the comparison of hypertension prevalence, however, were mixed. The HWOPs-1 study showed a prevalence of 36.1% compared with around 28% in a study that used the nationally representative WHO STEPwise data (Chege, 2016). Other studies conducted in urban areas, however, show much higher prevalence rates of 50% or higher (Mathenge et al., 2010). Additionally, 27.2% of the participants reported having been diagnosed with arthritis in the HWOPs-1 study, which shows a higher prevalence than that reported using the WHO SAGE Wave 1 study at 19.9% for women and 14.1% for men (Macia et al., 2016). The prevalence of other NCDs such as cancer and chronic lung disease showed a very low prevalence of 2.4% and 2.0%, respectively (Brennan-Olsen et al., 2017).

**Table 4.** Comparison of Diabetes and Hypertension Prevalence with Other Studies in Africa

Noncommunicable chronic condition	Prevalence from HWOPs-1(%)	Other studies in Africa		
Diabetes	11.7%	Mekelle City, Ethiopia: 13.6% (Gebreegziabiher, Belachew, & Tamiru, 2020)	Kenya: 14.6% (STEPwise National study; Chege, 2016)	Nakuru, Kenya: 6.6% (Mathenge, Foster, & Kuper, 2010)
Hypertension	36.1%	Kenya: 27.8% (STEPwise National study; Chege, 2016)	Nakuru, Kenya: 50.1% (Mathenge, Foster, & Kuper, 2010)	Dakar, Senegal: 58.8% (Macia, Gueye, & Duboz, 2016)
Arthritis	27.2%	Ghana: 19.1%; South Africa: 29% (WHO SAGE Wave1; Brennan-Olsen et al., 2017)		

*Notes*: SAGE = Study on Global AGEing and adult health; STEPwise = WHO STEPwise approach to Non-Communicable Diseases risk factors; WHO = World Health Organisation; HWOPs-1 = Health and Well-being of Older Persons-1.

Table 5 shows the correlation matrix between various health indicators with vision impairment and difficulty with feeding or chewing food. The composite scales to assess functionality, disability, and QoL showed a very strong correlation with each other and with the single global health and QoL measure. The two indicators, vision impairment and difficulty with feeding which are known to have a great impact on QoL and well-being showed strong association with the single item -global health status and global QoL. There was however a very weak correlation between vision impairment and difficulty with feeding with the three composite scales, that is, functionality scale, disability scale, and composite QoL scale.

**Table 5.** Correlation Between Health Indicators with Visual Impairments and Difficulty with Feeding

Health and well-being indicators	Visual impairment	Difficulty feeding and chewing	Single item overall health rating	Single item overall QoL rating	Functionality score	Disability score	QoL score
Visual impairment	1						
Difficulty feeding and chewing	–0.105^**^	1					
Single item overall health rating	0.246^***^	–0.141^***^	1				
Single item overall QoL rating	0.201^***^	–0.184^***^	0.486^***^	1			
Functionality score	0.011	0.019	–0.045	0.041	1		
Disability score	–0.001	–0.028	0.049	–0.051	–0.913^***^	1	
QoL score	0.047	0.0378	–0.020	0.052	0.703^***^	–0.747^***^	1

*Notes*: QoL = quality of life.

^*^ *p* < .05.

^**^ *p* < .01.

^***^ *p* < .001.

The correlation between the health measures and selected socioeconomic status is shown in Table 6. The socioeconomic status indicators used are ever attended school, level of education, type of employment prior to age 50 years, and source of livelihood. Education showed the strongest correlation with all the health measures, and type of employment showed a weak albeit statistically significant correlation with the health measures. The correlation between the source of livelihood with the health measures was however very low and not statistically significant.

**Table 6.** Correlation Between Health Indicators and Socioeconomic Variables

Socioeconomic indicators	Ever attended school	Level of education	Employment status prior to 50 years	Main source of livelihood	Functionality	Disability	QoL items
Ever attended school	1						
Level of education	0.924^***^	1					
Employment status prior to 50 years	0.138^***^	0.161^***^	1				
Main source of livelihood	0.096	0.054	0.059	1			
Functionality	–0.023	–0.265^***^	–0.082^*^	0.009	1		
Disability	0.019	0.332^***^	0.087^*^	0.013	–0.913^***^	1	
QoL items	0.037	–0.247^***^	–0.027	0.025	0.703^***^	–0.747^***^	1

*Notes*: QoL = quality of life.

^*^ *p* < .05.

^**^ *p* < .01.

^***^ *p* < .001.

The correlation between the measures of well-being and the risk factors for NCDs is shown in Table 7. The risk factors for NCDs used are: ever-used tobacco, ever-used alcoholic beverages, increase in heart rate due to work, fruit servings per day, vegetable servings per day, and the daily average number of meals consumed. The measures of well-being employed were the current health self-rating, overall state of happiness, and overall satisfaction with life. All three health measures were negatively correlated with the three risk factors for NCDs (i.e., use of tobacco, alcohol, and increased heart rate due to work) and positively correlated with positive health behaviors of number of fruits and vegetables and average meals per day although they were all weak correlations but at various levels of statistical significance.

**Table 7.** Correlation Between Measures of Well-being and Risk Factors for Noncommunicative Diseases

Health and well-being indicators	Current Health self-rating	Overall state of happiness	Overall satisfaction with life	Ever used tobacco	Ever taken alcoholic beverages	Increase in heart rate due to work	Fruit servings per day	Vegetable servings per day	Average meals per day
Current health self-rating	1								
Overall state of happiness	0.370^***^	1							
Overall satisfaction with life	0.425^***^	0.666^***^	1						
Ever used tobacco	–0.127^**^	–0.0132	0.003	1					
Ever taken alcoholic beverages	–0.168^***^	–0.049	–0.048	0.619^***^	1				
Increase in heart rate due to work	–0.234^***^	–0.044	–0.063	0.106^**^	0.138^***^	1			
Fruit servings per day	0.067	0.036	0.109^**^	0.083^*^	0.132^**^	0.100^*^	1		
Vegetable servings per day	0.095^**^	0.044	0.090^*^	0.0498	0.055	0.072^*^	0.502^***^	1	
Average of meals per day	0.051	0.132^***^	0.177^***^	0.024	–0.039	0.088^*^	0.206^***^	0.160^***^	1

*Note*:

^*^ *p* < .05.

^**^ *p* < .01.

^***^ *p* < .001.

## Discussion and Implications

The Health and Wellbeing of Older Persons in Kenya (HWOPs-1) questionnaire was developed as a standardized instrument that can be used for periodic monitoring of the health and well-being of older adults 60 years or older in Kenya. This study assessed the psychometric properties of the questionnaire by analyzing the correlation of commonly used health indicators, multi-item scales for measuring performance on IADL, ADL, and QoL, and single-item indicators that measure overall health, life satisfaction, and happiness with factors known to be strongly associated with health outcomes. The analyses show the questionnaire to have very good internal consistency, validity, and reliability.

The results from the analyses for internal consistency had a Cronbach alpha value of 0.89 for the QoL subscale, 0.94 for disability subscale, and 0.97 for the functionality subscale. Results from the *t* test showed statistically significant differences between men and women across the three subscales with *t* = −7.91, *p* < .001 for functionality, *t* = −7.12, *p* < .001 for disability, and *t* = 0.94, *p* > .1 for QoL. The correlations for health indicators and socioeconomic variables and for measures of well-being and risk factors for NCDs were weak but statistically significant which ranged from *r* = -0.265 to *r* =  +0.502.

The findings from the analyses demonstrated very high internal consistency, validity, and reliability of the HWOPs-1 questionnaire. The results showed the expected correlation between the health measures and factors known to be associated with or to affect health and well-being. The HWOPs-1 questionnaire included an 18-item functionality scale, a 24-item disability scale, and an 8-item QoL scale; these scales were highly correlated with each other; however, they were not strongly correlated with other health and well-being indicators namely visual impairment and difficulty with feeding and chewing. Conversely, the single-item measures on overall health, life satisfaction, and happiness were more strongly correlated with factors known to be associated with health compared with the multi-item scores. Extensive research has gone into the development of multi-item scales or indicators to measure functional status and QoL (Bowling, 2005; Cunny & Perri III, 1991). Similarly, to previous studies (de Boer et al., 2004; DeSalvo et al., 2006; Wasson, 2019), the present study shows single-item measures to be powerful and reliable in assessing health status, overall well-being, and QoL. In order to reduce the burden and the cost of administering lengthy questionnaires for periodic data collection, shorter and single-item health and well-being indicators are recommended.

The health indicators assessed in the study showed the expected association with education. Studies have expounded on the intricate relationship between education and health and explored mediating factors such as economic, social, psychological and interpersonal as well as behavioral factors (Raghupathi & Raghupathi, 2020). The health indicators however showed non-significant correlations with sources of livelihood pointing to the need to identify other more critical factors to assess socioeconomic status among the similar population of older adults 60 years or older (Khalatbari-Soltani et al., 2020; O’Rand & Lynch, 2018; Steptoe & Zaninotto, 2020).

This study has demonstrated the validity and applicability of the HWOPs-1 questionnaire, however, there are a few limitations. First, the study findings on health status are self-reporting. Secondly, whereas various methods and procedures of ascertaining a questionnaire’s validity were followed, the piloting was conducted in only one out of the 47 counties in Kenya thus not capturing the geographical, environmental, social, and cultural variability in the country. Another limitation is that findings on health status may not be largely generalizable and can only hold true for the area of study. More extensive and country-representative studies will need to be carried out to provide more encompassing and comprehensive data.

The availability of the HWOPs-1 questionnaire for assessing the health and well-being of older persons marks a great milestone towards addressing the data gaps on older adults 60 years or older in Kenya. This study establishes that the psychometric properties of the health module have indicators that have been used in settings outside Africa to measure health and well-being of older adults and that are adaptable and reliable enabling comparability across space and across studies. The HWOPs-1 questionnaire was found to have valid items as demonstrated by the results of the factor analysis which showed higher factor loadings across the three subscales that were analyzed. The reliability and validity of the HWOPs-1 questionnaire were further confirmed by the measure of internal consistency, which showed a strong Cronbach’s alpha value ranging from 0.89 to 0.97. This implies that the tool is valid and acceptable for measuring the health status and well-being of older Kenyans. It provides a basis for future studies to delve into patterns, trends, and success of current interventions available for measuring the health and well-being of older adults. The HWOPs-1 study may be replicated in other regions of the country to provide comprehensive data on NCDs and other health and well-being indicators. Additionally, it is hoped that the HWOP questionnaire can be used to provide information to adequately inform the various national policies relating to aging and also inform the implementation of programs to improve the living standards of older adults 60 years or older in Kenya.
